# Field—Based Supercritical Fluid Extraction of Hydrocarbons at Industrially Contaminated Sites

**DOI:** 10.1100/tsw.2002.205

**Published:** 2002-04-19

**Authors:** Peggy Rigou, Steven John Setford, Selwayan Saini

**Affiliations:** Cranfield Centre for Analytical Science, Cranfield University at Silsoe, Silsoe, Bedfordshire, MK45 4DT, UK

**Keywords:** field-based, supercritical fluid extraction (SFE), Soxhlet, field-based solvent extraction, polynuclear aromatic hydrocarbon (PAH), benzene, toluene, ethylbenzene, xylene (BTEX)

## Abstract

Examination of organic pollutants in groundwaters should also consider the source of the pollution, which is often a solid matrix such as soil, landfill waste, or sediment. This premise should be viewed alongside the growing trend towards field-based characterisation of contaminated sites for reasons of speed and cost. Field-based methods for the extraction of organic compounds from solid samples are generally cumbersome, time consuming, or inefficient. This paper describes the development of a field-based supercritical fluid extraction (SFE) system for the recovery of organic contaminants (benzene, toluene, ethylbenzene, and xylene and polynuclear aromatic hydrocarbons) from soils. A simple, compact, and robust SFE system has been constructed and was found to offer the same extraction efficiency as a well-established laboratory SFE system. Extraction optimisation was statistically evaluated using a factorial analysis procedure. Under optimised conditions, the device yielded recovery efficiencies of >70% with RSD values of 4% against the standard EPA Soxhlet method, compared with a mean recovery efficiency of 48% for a commercially available field-extraction kit. The device will next be evaluated with real samples prior to field deployment.

## INTRODUCTION

The presence of persistent organic pollutants in groundwaters from contaminated sites is a significant problem and requires a coordinated approach, requiring not only the consideration of the presence and movement of the pollution in groundwaters, but also the source of these pollutants which may be within contaminated soils, landfills, and sediments. This consideration should be examined alongside the growing trend of field-based characterisation of contaminated sites for cost reasons and to allow rapid on-site decisions to be made regarding site treatment and remediation[1].

As in the laboratory, a successful site-based characterisation protocol demands appropriate sample extraction prior to analysis. Soxhlet extraction is perhaps the most widely accepted U.S. Environmental Protection Agency- (EPA-) approved laboratory method for the removal of hydrocarbon contaminants from soil samples, but it is ill-suited to the field for practical reasons (elaborate methods, long extraction times, delicate glassware, and safety issues). There are also associated issues regarding disposal of the large amounts of waste solvents generated by this method. More field-amenable methods include simple solvent extraction, but [these methods] recover only those species loosely bound to the sample matrix, providing an incomplete picture of the extent of contamination[1].

The limitations of these conventional methods have led to the consideration of alternative extraction procedures. Supercritical fluid extraction (SFE) is a well-established EPA-approved method for the laboratory-based extraction of analytes from soils[2,3]; however, to date there have been no reports regarding the development of dedicated field-based SFE devices (the nearest equivalent being the deployment by Bøwadt et al.[1] of a cumbersome laboratory-based system within an all-terrain vehicle). This absence of field-based systems is surprising given the essential simplicity of SFE (controlled passage of a heated fluid under pressure through a soil sample) coupled to its speed, efficiency (comparable to Soxhlet), robustness, and minimal solvent consumption; therefore, the aim of this study is to develop and optimise a field-portable SFE system for the extraction of hydrocarbon contaminants from soils. Benzene, toluene, ethylbenzene, and xylene (BTEX) and polynuclear aromatic hydrocarbons (PAHs) were chosen since they are toxic and carcinogenic and widespread in the environment, and they represent a broad range of volatilities and physico-chemical properties[4]. A state-of-the-art design of back pressure regulator (BPR) was assessed as a means of eliminating the problem of restrictor blockage that has been such a significant problem to SFE practitioners over many years[4].

## EXPERIMENTAL METHODS AND PROCEDURES

### Soil Spiking

A solution containing 100 µg $ml^{-1}$ each of benzene, toluene, ethylbenzene, *s*-, *m*-, *p*-xylene (environmental standards for EPA methods, 5000 mg, neat; Supelco, Dorset, U.K.), and naphthalene, acenaphthalene, fluorene, phenanthrene, anthracene, fluoranthrene, pyrene, chrysene, and benzo[*a*]pyrene (Sigma-Aldrich, Poole, U.K.) was prepared in dichloromethane (DCM, Sigma-Aldrich). A 2.5-ml volume was spiked onto 10 g of a standard soil (6.8% clay, 82% sand, 1.9% carbon) and packed into a 10-ml capacity stainless steel extraction vessel (ISCO, Inc., Lincoln, NE, U.S.) such that the spiked analytes were eluted through the vessel during extraction[2]. Samples were extracted immediately after spiking.

### Extraction Procedure for Lab-Based and Field-Based SFE Systems

Two SFE systems were optimised for the extraction of BTEX and PAHs. The laboratory-based system consisted of 260D and 100D syringe pumps (ISCO, Inc.) for $CO_2$ and modifier (methanol)

addition, respectively; both pumps required water-cooling and were controlled using a 260D control box. The extraction vessel was heated in a GC oven. An adjustable restrictor (ISCO, Inc.) allowed manual control of the fluid flow through the system. The temperature of the restrictor valve body was set to that of the oven with the tip having a bias of +35°C to minimise restrictor blockage[4]. The field-based system consisted of a PU-1580 $CO_2$ dual reciprocating pump (JASCO, Great Dunmow, Essex, U.K.) with Peltier electrical cooling, compact oven, and a BPR. The BPR was controlled from the pump, allowing precise control of system pressure and flow rate. Analytes were collected in a glass vial containing 10 ml of dichloromethane or methanol cooled to 5°C prior to extraction[2]. The complete system is shown in Fig. 1.

## Soxhlet Extraction

Soxhlet extractions (EPA method SW-846 #3540C) of 10 g of spiked soils were performed for 20 h with 350 ml of DCM and concentrated to a final volume of 10 ml.

## Field-Based Solvent Extraction

Spiked soil samples (10 g) were extracted using a commercially available field-based sample extraction kit supplied by SDI (Strategic Diagnostic, Inc., Hants, U.K.) by shaking with 10 ml of 75% v/v methanol, settling for 5 min, filtering the extract, and reconstituting to 10 ml.

## Extract Analysis

*Chromatograph*: Perkin Elmer 8000 gas chromatograph equipped with a PE Nelson 900 series interface and a flame ionisation detector with He carrier gas and $H_2$/air detector gas. *BTEX analysis*: 30 m × 0.32 mm (1 µm film) DB-5 column (J&W Scientific, Inc., U.S.); injector and detector temp: 250°C and 320°C, respectively; oven: 35°C for 1 min, ramped at 25°C $min^{-1}$ to 150°C and held until all analytes eluted. *PAH analysis*: 30 m × 0.25 mm (0.25 µm film) AT-5MS column (Alltech, Lancs., U.K.); injector/detector temp: 340°C; oven: 80°C for 1 min, ramped at 25° C $min^{-1}$ to 160°C, then at 25°C $min^{-1}$ to 325°C and held until all analytes eluted.

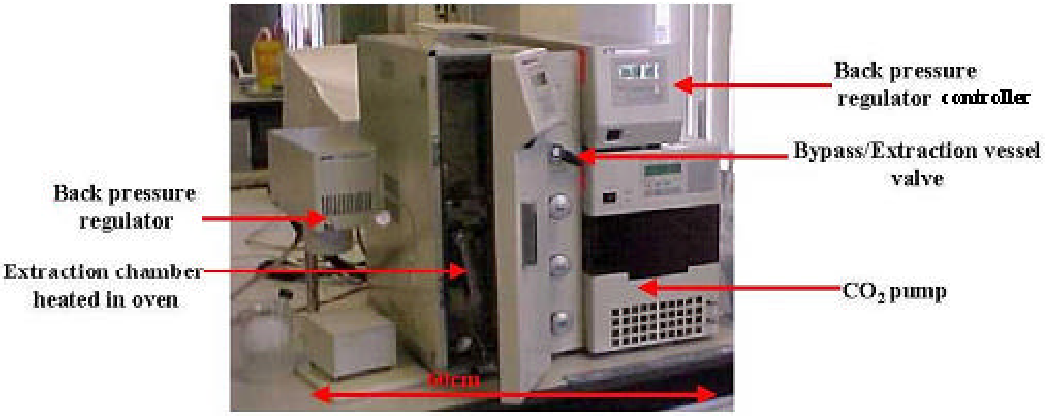

**FIGURE 1.** Field-based SFE system with, from the right to the left, the $CO_2$ pump, oven, and back pressure regulator.

## RESULTS AND DISCUSSION

### Collection Solvent

Whilst both DCM and methanol proved suitable collection solvents for both BTEX and PAHs, the latter solvent was favoured for reasons of reproducibility and reduced solvent volatilisation. Furthermore, methanol is more compatible with the envisaged field-based analytical (immunoassay) procedure, requiring only simple dilution in aqueous buffer, rather than the addition of cosolvent (e.g., propan-2-ol) to engender compatibility with the aqueous-phase.

### SFE Conditions

The five key extraction variables of temperature (A), pressure (B), extraction time (C), modifier addition (D), and fluid flowrate (E) were evaluated in this study by the factorial analysis procedure[5]. The high/low values for each variable were

**BTEX:** A: 100/50°C; B: 35.5/13.8 MPa; C: 30/15 min; D: 1%/0% v/v methanol; E: 0.2/0.8 ml $min^{-1}$;
**PAH:** A: 120/60°C; B: 35.5/13.8 MPa; C: 30/15 min; D: 1%/0% v/v methanol; E: 0.2/0.8 ml $min^{-1}$.

The statistically verified optimum conditions for extraction were

**BTEX**: A: 50°C; B: 13.8 MPa; C: 30 min; D: 0% v/v methanol; E: 0.8 ml $min^{-1}$;
**PAH:** Spiked PAHs required a two-step extraction due to their widely varying physico-chemical properties, with the more volatile PAHs (naphthalene-phenanthrene) extracted as per step 1 (A: 60°C; B: 13.8 MPa; C: 15 min; D: 0% v/v methanol; E: 0.8 ml $min^{-1}$), and the less volatile PAHs (anthracene-benzo[*a*]pyrene) extracted as per step 2 (A: 120°C; B: 35.5 MPa; C: 30 min; D: 0% v/v methanol; E: 0.8 ml $min^{-1}$), these findings being consistent with other reported studies[2,6,7,8]. Under these optimised conditions, extraction efficiencies of >70% were achieved for all analytes except benzene, presumably due to its high vapour pressure.

### Comparison of the Dual-Reciprocating Pump-BPR and Syringe Pump-Regulator SFE Systems

Both systems performed similarly for the extraction of BTEX and PAH compounds from spiked soil samples; however, the dual-reciprocating pump-BPR system exhibited a number of benefits over the syringe pump-regulator system that make it more amenable to field usage:

- The system is easily set up via HPLC-type fittings to a suitable $CO_2$ supply (5-kg cylinder for field-based system).
- Simultaneous pressurisation and pumping of supercritical fluids eliminates the need for complete system depressurisation and refilling/pressurisation of the syringe pump, a time-consuming process.
- Peltier-controlled cooling eliminates the need for an associated water-jacket cooling system.
- The system is compact and transportable (total system dimensions: 55(W) × 43(D) × 31(H) cm, 35 kg).
- Standard SFE restrictor systems operate on the principle of flow restriction to control flowrate and pressure. The BPR unit operates by regulating fluid flow/pressure by means of a solenoid-driven oscillating needle valve. This approach greatly reduces the problem of blocking caused by particulates entering restrictor-type systems in that the needle opens/closes to a greater extent to maintain the desired flowrate/pressure.

- The BPR system is electronically controlled via the pump controller, eliminating problems associated with manual control of flowrate and offers improved extraction reproducibility (BPR: RSD = 3–4%; manual restrictor, RSD = 6–8%).

## Comparison of SFE Against Soxhlet and Field-Based Solvent Extraction

The extraction performance of the Soxhlet, SFE, and field-based (SDI) solvent extraction methods are shown in Table 1. All extraction efficiencies were compared against the EPA standard Soxhlet method; therefore all Soxhlet extraction efficiencies were equivalent to 100% recovery of analytes and the SFE and SDI extraction values proportionally recalculated. Efficiencies >100% thus indicate a more efficient extraction than Soxhlet. SFE is clearly a preferential method for the extraction of very volatile compounds that are otherwise lost by Soxhlet; however, for less-volatile compounds, Soxhlet showed similar or higher extraction efficiencies than SFE, consistent with other reports[8,9]. Preliminary studies indicate the lower recoveries of the less-volatile PAHs will be improved by modifier addition[2,10]. The commercially available field solvent extraction method yielded the poorest extraction efficiency (48% mean, vs. 82% for SFE and 100% for Soxhlet).

**TABLE 1**
**Comparison of Extraction Efficiencies (%) of Soxhlet, SFE, and Field-based (SDI) Solvent Extraction for Recovery of BTEX and PAH Compounds from Spiked Soil Samples**

| | Extraction efficiency ± RSD | | |
|---|---|---|---|
| **Compounds** | **Soxhlet** | **SFE** | **SDI** |
| benzene | 100 ± 18 | 137 ± 10 | 98 ± 10 |
| toluene | 100 ± 13 | 152 ± 6 | 67 ± 6 |
| ethylbenzene | 100 ± 5 | 94 ± 5 | 49 ± 6 |
| *m*-, *p*-xylene | 100 ± 4 | 88 ± 4 | 32 ± 4 |
| *s*-xylene | 100 ± 4 | 83 ± 4 | 26 ± 3 |
| naphthalene | 100 ± 6 | 76 ± 4 | 54 ± 6 |
| acenaphthalene | 100 ± 4 | 69 ± 4 | 49 ± 3 |
| fluorene | 100 ± 16 | 75 ± 3 | 53 ± 3 |
| phenanthrene | 100 ± 6 | 73 ± 3 | 50 ± 3 |
| anthracene | 100 ± 11 | 76 ± 2 | 52 ± 3 |
| fluoranthrene | 100 ± 4 | 65 ± 3 | 43 ± 2 |
| pyrene | 100 ± 8 | 64 ± 4 | 42 ± 2 |
| chrysene | 100 ± 10 | 45 ± 4 | 27 ± 3 |
| Benzo[*a*]pyrene | 100 ± 17 | 49 ± 3 | 33 ± 1 |

*Note:* All extraction efficiencies are compared against EPA Soxhlet method SW-846 #3540C; thus, all Soxhlet efficiencies are recorded as 100% ± % SD. Extraction efficiencies of >100% indicate a more efficient extraction of analyte compared with the equivalent Soxhlet extraction.

SD = Standard deviation
SFE = Supercritical fluid extraction
SDI = Strategic Diagnostics Inc. Solvent Extraction Kit
BTEX = benzene, toluene, ethylbenzene, and xylene
PAH = polyaromatic hydrocarbons

## SFE: Benefits and Drawbacks

For field-based deployment, SFE has a number of advantages over Soxhlet extraction. Following method development, it is relatively simple to set-up, requiring only simple sample homogenisation (e.g., grinding by mortar and pestle) before it is loaded into an extraction vessel and a pre-programmed extraction procedure initiated. According to EPA methods SW-846 #3560 and #3561, SFE can tolerate up to 50% moisture in the sample, although premixing with diatomaceous earth may improve extraction efficiencies for certain samples. SFE uses a fraction of the solvent required by Soxhlet (10 ml vs. 300 ml), thereby preventing the creation of a significant waste disposal issue. With an extraction time of 30 min, it is also more rapid than Soxhlet (typically 16–24 h). Whilst the field-based solvent extraction method is more rapid and simple than SFE, it yields questionable data due to the poor extraction efficiencies obtained. The main drawback of SFE is initial equipment cost, but this should be weighed against the benefits of field-based SFE where, due to its efficiency, large numbers of samples can be processed on-site in a day, with minimum solvent disposal costs.

## CONCLUSION

Two laboratory-based SFE systems, one of which will be further developed for field-based deployment, were optimised for extraction of BTEX and PAH compounds from spiked soil samples. Extraction recoveries were shown to be within 70% of the EPA standard Soxhlet method, but much higher than a commercially available field-based solvent extraction test kit. As a field-based extraction procedure, SFE is an attractive technique primarily because it is relatively simple and rapid (~30 min), generates minimum solvent waste, and yields high extraction efficiencies. Further work will focus on developing the compact reciprocating pump-BPR system as a field-based extraction tool and will consider sample moisture content issues. This system will be optimised for the extraction of real-world samples and compared with Soxhlet and field-based solvent extraction methods using a standard laboratory-based chromatographic method EPA approved and immunoassay test kits. The device will then be evaluated in the field.

## ACKNOWLEDGEMENT

This paper was presented at the CSIC/ESF workshop Analysis, Toxicity, and Biodegradation of Organic Pollutants in Groundwater from Contaminated Land, Landfills, and Sediments, Barcelona, Spain, 8–10 November, 2001. The support of the European Commission under the PURE Project (EVK1-CT1999-00030) is gratefully acknowledged.

## References

1. Bøwadt, S., Mazeas, L., Miller, D.J., and Hawthorne, S.B. (1997) Field-portable determination of polychlorinated biphenyls and polynuclear aromatic hydrocarbons in soil using supercritical fluid extraction. *J. Chromatogr. A* **785**, 205–217.
2. Reindl, S. and Höfler, F. (1994) Optimization of the parameters in supercritical fluid extraction of polynuclear aromatic hydrocarbons from soil samples. *Anal. Chem.* **66**, 1808–1816.
3. Yang, Y., Hawthorne, S.B., and Miller, D.J. (1995) Comparison of sorbent and solvent trapping after supercritical fluid extraction of volatile petroleum hydrocarbons from soils. *J. Chromatogr. A* **699,** 265–276.
4. Burford, M.D, Hawthorne, S.B, and Miller, D.J. (1993) Extraction rates of spiked versus native PAHs from heterogeneous environmental samples using supercritical fluid extraction and sonication in methylene chloride. *Anal. Chem.* **65**, 1497–1505.
5. Davies, O.C. (1978) *The Design and Analysis of Industrial Experiments*. 2nd ed. Oliver and Boyd, Tweeddale Court, Edinburgh.

6. Hawthorne, S.B., Grabanski, C.B., Martin, E., and Miller, D.J. (2000) Comparison of soxhlet extraction, pressurized liquid extraction, supercritical fluid extraction and subcritical water extraction for environmental solids: recovery, selectivity and effects on sample matrix. *J. Chromatogr. A* **892,** 421–433.
7. Hawthorne, S.B., Galy, A.B., Schmitt, V.O., and Miller, D.J. (1995). Effect of SFE flow rate on extraction rates, classifying samples extraction behaviour. *Anal. Chem.* **67**, 2723–2732.
8. Bøwadt, S. and Hawthorne, S.B. (1995) Supercritical fluid extraction in environmental analysis. *J. Chromatogr. A* **703(5)**, 549–571.
9. Berset, J.D, Ejem, M., Holzer, R., and Lisher, P. (1999) Comparison of different drying, extraction and detection techniques for the determination of priority polycyclic aromatic hydrocarbons in background contaminated soil samples. *Anal. Chimica Acta* **383**, 263–275.
10. Langenfeld, J.J., Hawthorne S.B., Miller, D.J., and Pawliszyn, J. (1994) Role of modifiers for analytical-scale supercritical fluid extraction of environmental samples. *Anal. Chem.* **66**, 909–916.
